# The Heart‐To‐Single Vertebra Ratio for Assessing the Cardiac Silhouette Size in Cats

**DOI:** 10.1111/vru.70042

**Published:** 2025-05-05

**Authors:** Dario Costanza, Adelaide Greco, Diego Piantedosi, Erica Castiello, Pierpaolo Coluccia, Camilla Sangiuliano, Luigi Navas, Leonardo Meomartino

**Affiliations:** ^1^ Interdepartmental Centre of Veterinary Radiology, University of Napoli "Federico II" Napoli Italy; ^2^ Department of Veterinary Medicine and Animal Productions University of Napoli "Federico II" Napoli Italy

**Keywords:** cardiology, feline, thoracic radiography, vertebral heart scale, vertebral heart score

## Abstract

The heart‐to‐single vertebra ratio (HSVR) has been proposed as a simple, quick, and reliable radiographic method to assess cardiac silhouette dimensions in dogs. The HSVR shows excellent agreement with the vertebral heart size (VHS), and it can also be accurately determined in dogs with vertebral abnormalities affecting the thoracic spine. This retrospective, single‐center, method‐comparison, observer‐agreement study investigated the reliability of the HSVR in cats. Three observers retrospectively evaluated anonymized right‐lateral thoracic radiographs obtained over a set period of time. Exclusion criteria included the presence of thoracic spine alterations and the inability to outline the cardiac silhouette. The HSVR was calculated by dividing the sum of the cardiac long and short axes by the length of each vertebral body from T4 to T8. Images of 101 cats of different breeds met the inclusion criteria. Lin's concordance correlation coefficient and the relative 95% confidence interval (CI) revealed that the HSVR^T6^ showed the best agreement with the VHS (0.95; 95% CI: 0.91–0.97). Bland–Altman plots showed low bias (−0.023 ± 0.19; limits of agreement = −0.39 to 0.35) between the HSVR^T6^ and the VHS, with low mean absolute error (0.14; 95% CI: 0.12–0.17) between the two methods. The intraclass correlation coefficients, evaluated on 20 cats, demonstrated excellent interobserver agreement (0.95–0.96; *p* < .001) and good to excellent intraobserver agreement (0.75–0.94; *p* < .001) for all HSVRs. The results of this study confirmed that the HSVR is a simple, quick, and reliable alternative to the VHS also in cats.

## 1 Introduction

Although echocardiography is the gold standard modality for detecting cardiac disease, radiography still plays a fundamental role [1, 2]. Indeed, radiography is widely used in veterinary clinical practice because it is a quick and affordable imaging method that is easy to perform. It also allows concurrent evaluation of other thoracic structures, including the lung parenchyma and pulmonary airways, whose primary pathologies may present with clinical signs similar to those of congestive heart failure, including an increased respiratory rate, breathlessness, cough, and cyanosis [3, 4]. The radiographic diagnosis of cardiac disease is based on the recognition of alterations in the shape and size of the cardiac silhouette, pulmonary vessels, and possible accompanying pulmonary changes consistent with a condition of congestive heart failure, such as pulmonary edema or pleural effusion [3, 4, 5]. However, it may be difficult to detect cardiomegaly because of its variable features in healthy cats and dogs and a possible overlap between healthy and diseased subjects [5]. This variability is particularly challenging for nonspecialists and may hinder the detection of cardiac disease or result in an overestimation of normal cardiac dimensions [6]. Consequently, in recent decades, various methods have been proposed to simplify the radiographic determination of altered cardiac silhouette dimensions [7, 8, 9, 10]. However, those methods were strongly influenced by poorly controllable variables, such as the respiratory phase and magnification of the intercostal spaces [4]. In 1995, Buchanan and Bücheler proposed the vertebral heart size (VHS) [11], a method to easily determine the cardiac silhouette dimensions in dogs by transposing the cardiac long and short axes (LA and SA, respectively) over the vertebral column and converting their lengths into vertebral units (v) to the nearest 0.1 v. Several years later, Litster and Buchanan evaluated the VHS in cats and proposed an upper cut‐off value of 8.0 v as the limit for a normal‐sized heart [12]. Subsequent studies reported similar cut‐off values for the VHS in different breeds and obese patients and found that, unlike in dogs, the dimensions of the normal feline heart are generally barely affected by breed and body condition score [4, 13–16]. Multiple studies also investigated the diagnostic accuracy of the VHS in detecting cardiac disease [2, 4, 5, 17, 18]. One limitation is the difficulty of correctly determining the VHS in subjects with thoracic vertebral column alterations, including spondylosis deformans and decreased intervertebral disc space [19, 20]. Furthermore, based on our clinical experience, we believe that lordosis of the thoracic vertebral column, which feline patients tend to assume during thoracic radiography examinations, could be an additional limitation in obtaining the VHS. Indeed, these alterations may result in the VHS being overestimated, which would limit its clinical usefulness.

Recently, the heart‐to‐single vertebra ratio (HSVR) method was proposed as a valid alternative to the VHS [19]. In this new method, the sum of the cardiac LA and SA is divided by the length of a single thoracic vertebra of the spine between the fourth and eighth thoracic vertebra, with both measurements expressed in the same unit of measurement. The resulting HSVR is a dimensionless, pure number. The advantages of the HSVR over the VHS include the ability to compare cardiac dimensions with the vertebral column, even in patients with thoracic vertebral alterations. Furthermore, the newly described method showed similar inter‐ and intraoperator agreement to VHS and is quicker to perform because it does not require transposition of the cardiac axes over the thoracic vertebral column and spanning the LA and SA lengths in vertebral units.

The present study sought to test the HSVR method in cats and establish which HSVR value shows the greatest agreement with the VHS. We hypothesized that the HSVR is an easy‐to‐perform, reliable radiographic method for straightforward evaluation of the cardiac silhouette dimensions in cats, as already demonstrated for dogs. Our secondary aims included assessing the intra‐ and interobserver agreement for three observers with different levels of experience.

## 2 Materials and Methods

### 2.1 Selection and Description of Subjects

This single‐center, retrospective, method comparison, observer‐agreement study was approved by the Clinical Ethical Review Board of the University of Napoli “Federico II” (PG/2024/0097859). The electronic records of feline patients referred to the Interdepartmental Centre of Veterinary Radiology of the University of Napo “Federico II” in the specified period of January 2019 to December 2023 were reviewed, and the images were retrieved from the picture archiving and communication system (dcm4chee‐arc‐light version 5.11.1; http://www.dcm4che.org). The radiographic studies were performed for diverse clinical reasons, including assessing respiratory conditions, screening for cardiac disease, preanesthetic assessment, and exclusion of metastases. All radiographic examinations were obtained using a computed radiography or direct digital radiography system (Agfa CR‐30 and Agfa DR‐14e, respectively; Agfa HealthCare, Mortsel, Belgium). Both systems used the same focused Potter–Bucky grid and a set focus‐to‐film distance of 100 cm. All radiographic examinations in this study included the right‐lateral view, which was used to assess cardiac silhouette dimensions. Images were excluded for the following reasons: (1) presence of alterations affecting the thoracic vertebral column or the sternum (e.g., spondylitis, discitis, spondylosis deformans, reduced intervertebral disc space, pectus excavatum); (2) excessive thoracic spine lordosis; (3) inability to correctly visualize the cardiac silhouette (e.g., pleural effusion, increased pulmonary opacity over the cardiac silhouette, cardiac neoplasm, or mediastinal masses); (4) excessive thoracic rotation or technical errors (e.g., overexposure, underexposure, or motion artifacts); and (5) skeletal immaturity.

### 2.2 Data Recording and Analysis

The patient details, including breed, sex, weight (in kilograms), and age (in years), were retrieved from the clinical records of each cat. The decision on whether to include or exclude radiographs was made by one author (D. C.), a veterinarian with a PhD in veterinary diagnostic imaging and a first‐year resident of the European College of Veterinary Diagnostic Imaging. The sample size necessary for the inter‐ and intraobserver agreement was determined based on a priori power analysis (G*Power, v. 3.1.9.6, Heinrich‐Heine‐Universität Düsseldorf, Germany). The *F*‐tests family was selected, specifically the ANOVA: repeated measures within factors test. An effect size of 0.4, a significance level (*α*) = 0.05, and a power of 85% were applied for 3 groups and 2 measurements. The smaller set of radiographs required to assess the interobserver agreement was extracted from the final sample by one author (D. C.) using an online randomizer tool (https://www.randomizer.org), and the radiographs in DICOM format, were anonymized by the same author (D. C.) before being submitted to three observers with different levels of experience. Observer 1 (L. M.), a professor of veterinary radiology with a PhD and over 27 years of experience; Observer 2 (A. G.), a professor of veterinary radiology with a PhD and over 10 years of experience; and Observer 3 (D. P.), a professor of veterinary internal medicine with a PhD and over 17 years of experience in veterinary cardiology. Two months later, to assess intraobserver agreement, the same author (D. C.) re‐randomized the same smaller sample of radiographs and re‐submitted them to the observers. Each observer independently measured the parameters required to evaluate the inter‐ and intraobserver agreement by following the same written and illustrated instructions provided in a PDF file. The observers were blinded to the results reported by the other observers. Finally, the three observers measured the parameters on all of the radiographs included in the final sample in a single joint session and reached a consensus for each measurement. All radiographic parameters were measured on the right‐lateral projection using the electronic caliper (“length” tool) in an open‐source DICOM viewer (Horos version 4.0.0, 64‐bit; Nimble Co., LLC d/b/a Purview, Annapolis, MD, USA; https://www.horosproject.org) on the same workstation (iMac 5K, 27‐inch; Apple Inc., Cupertino, CA, USA).

**FIGURE 1:**
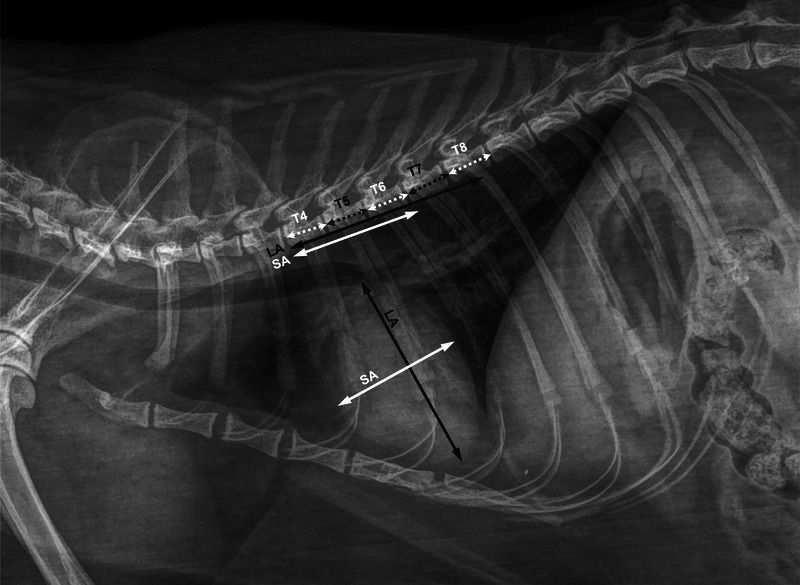
Representative right‐lateral thoracic radiographic image (73 kVp; 3.2 mAs) of a domestic short‐hair cat depicting the measurements of the length of every single vertebral body between T4 and T8, including the corresponding caudal intervertebral disc spaces (white and grey doubled‐headed dotted arrows, respectively, labeled T4–T8). The cardiac long axis (LA; doubled‐headed grey arrow) and short axis (SA; doubled‐headed white arrow) were measured as described by Litster and Buchanan [12]. In this method, the LA was traced from the ventral border of the carina to the cardiac apex, and the SA was traced perpendicular to LA at the point of the maximum width of the cardiac silhouette and then transposed ventrally to the column starting from the cranial endplate of T4.

Using the electronic caliper, the observers measured the length of the vertebral body of the fourth (T4), fifth (T5), sixth (T6), seventh (T7), and eighth (T8) thoracic vertebrae, including the respective caudal intervertebral disc space. Then, the LA and SA were measured as described by Litster and Buchanan (Figure 1) [12]. The LA was defined as the axis traced from the apex to the base of the cardiac silhouette, where it intersected the tracheal bifurcation. The SA was defined as the line perpendicular to the LA at the point of the maximum width of the cardiac silhouette. The two axes were transposed over the thoracic vertebrae to calculate the VHS, starting from the cranial endplate of T4. The VHS was then obtained, spanning the cardiac axis length as the number of thoracic vertebrae and rounding to the nearest 0.1 v. As previously described for dogs [19], HSVR was obtained by dividing the sum of the cardiac axes (LA + SA) by the length of the vertebral body of T4, T5, T6, T7, and T8, including the respective caudal intervertebral disc space, yielding HSVR^T4^, HSVR^T5^, HSVR^T6^, HSVR^T7^, and HSVR^T8^, respectively. Each result was rounded to the first decimal place and recorded by each operator in a separate electronic spreadsheet (Microsoft Excel version 16.81 2024; Microsoft Corp., Redmond, WA, USA).

### 2.3 Statistics

All of the measurements were de‐anonymized and collated in a single electronic spreadsheet before one author (D. C.), who was not previously involved in performing the measurements, performed the statistical analyses. The same author, who had training in statistics during the PhD course, performed the statistical analysis using commercial statistics software (IBM SPSS Statistics, version 29.0.1.0 for MacOS; IBM Corp, Armonk, NY, USA; GraphPad Prism, version 10.1.0(264); GraphPad Software LLC, Boston, MA, USA; MedCalc version 19.2.6; MedCalc Software Ltd, Ostend, Belgium). Descriptive statistics were calculated for age, weight, and breed. The normality of data was assessed using the Shapiro–Wilk test. Continuous data are reported as the mean ± standard deviation (SD) or as the median (range), depending on the distribution. The concordance between the HSVR and VHS values was evaluated based on the measurements taken by the three observers in the joint session using Lin's concordance correlation coefficient (CCC) along with the corresponding 95% confidence interval (95% CI) [21]. The strength of the agreement was scored using the cut‐off values proposed by McBride, analyzed using Bland–Altman plots, and by calculating the relative limits of agreement (LoA) [22, 23]. The error between the VHS and HSVR values was estimated using the mean absolute error (MAE). The repeated measurements of the second, smaller sample of radiographs were used to assess the intra‐ and interobserver agreement using intraclass correlation coefficients (ICC), with a two‐way mixed‐effects model for single measurements or single observers accordingly, and absolute agreement [24]. The relative 95% CIs were also calculated. The ICC results were interpreted following Koo and Li [24], where ICC values of <0.5 indicate poor reliability, 0.5 to <0.75 indicate moderate reliability, 0.75 to ≤0.9 indicate good reliability, and >0.9 indicate excellent reliability. In all analyses, *p* <.05 was considered statistically significant.

## 3 Results

Thoracic radiography was performed on 305 cats during the specified study period. After applying the exclusion criteria, right lateral radiographs of 101 cats (43 spayed females, 34 castrated males, 12 intact females, and 12 intact males) were included in the final sample. The power analysis determined that a sample size of 18 cats was required to assess both inter‐ and intraobserver agreement. Consequently, 20 radiographs were randomly selected from the final sample for this aim. The exposure settings (kilovoltage peak, milliamperes, and seconds) varied according to the cat's size and the body condition score. The median age was 8 years (range 1–18 years), and the mean ± SD body weight was 4.7 ± 1.1 kg. The breeds included domestic short‐hair (*n* = 75), domestic long‐hair (*n* = 16), Persian (*n* = 5), Maine Coon (*n* = 2), and one each of Ragdoll, Siamese, and Scottish straight.

**FIGURE 2:**
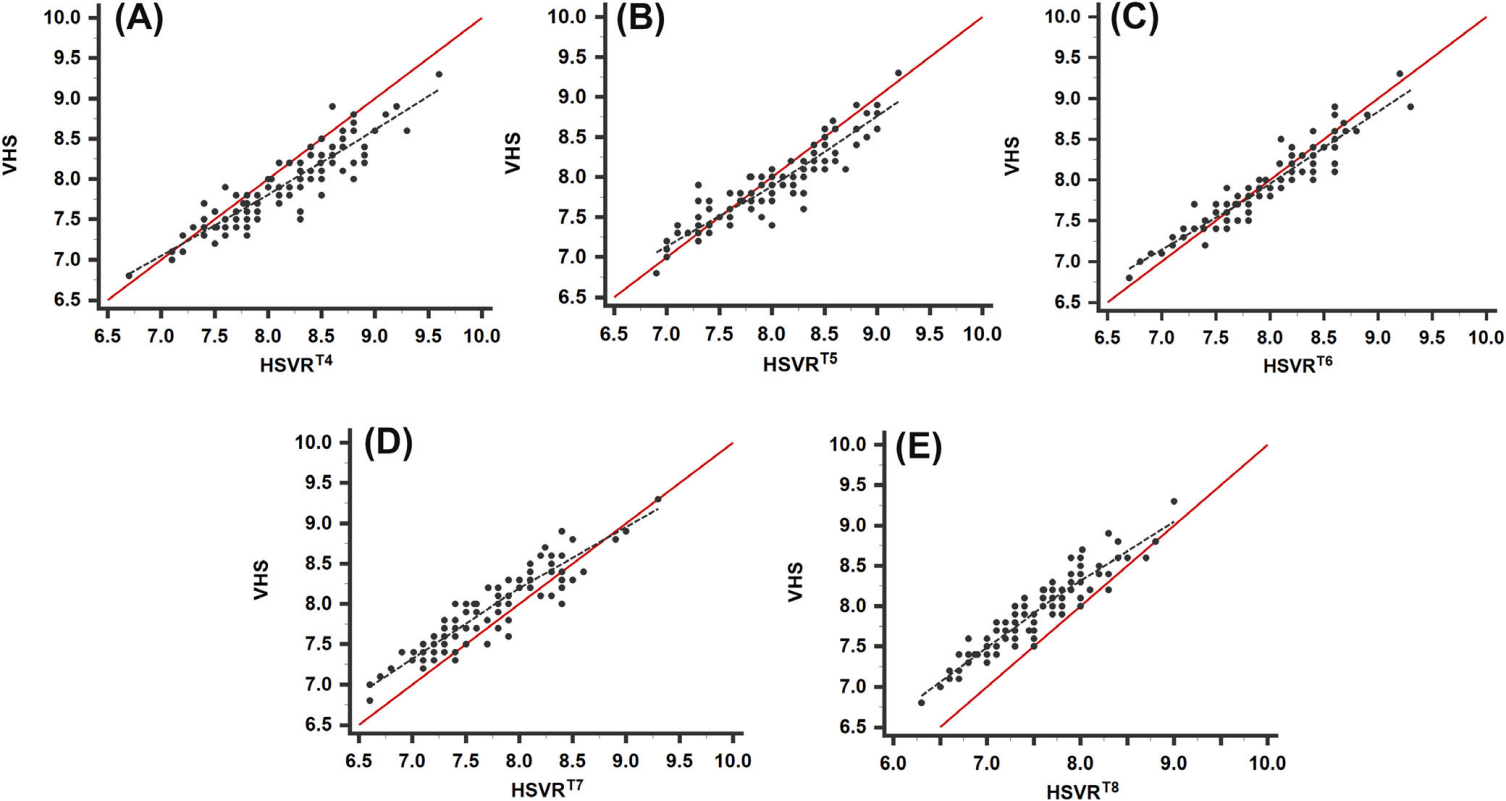
Lin's concordance correlation coefficient graphs comparing the VHS with HSVR determined using (A) T4, (B) T5, (C) T6, (D) T7, and (E) T8. The *y*‐axes show the VHS, and the *x*‐axes show the HSVR. The solid red lines represent perfect agreement (i.e., the ideal condition where HSVR equals VHS). The dashed black lines represent the estimated least squares lines (i.e., the deviations from agreement). HSVR^T^ *^n^*, heart‐to‐single vertebra ratio determined using the *n*th thoracic vertebra; VHS, vertebral heart size.

**FIGURE 3:**
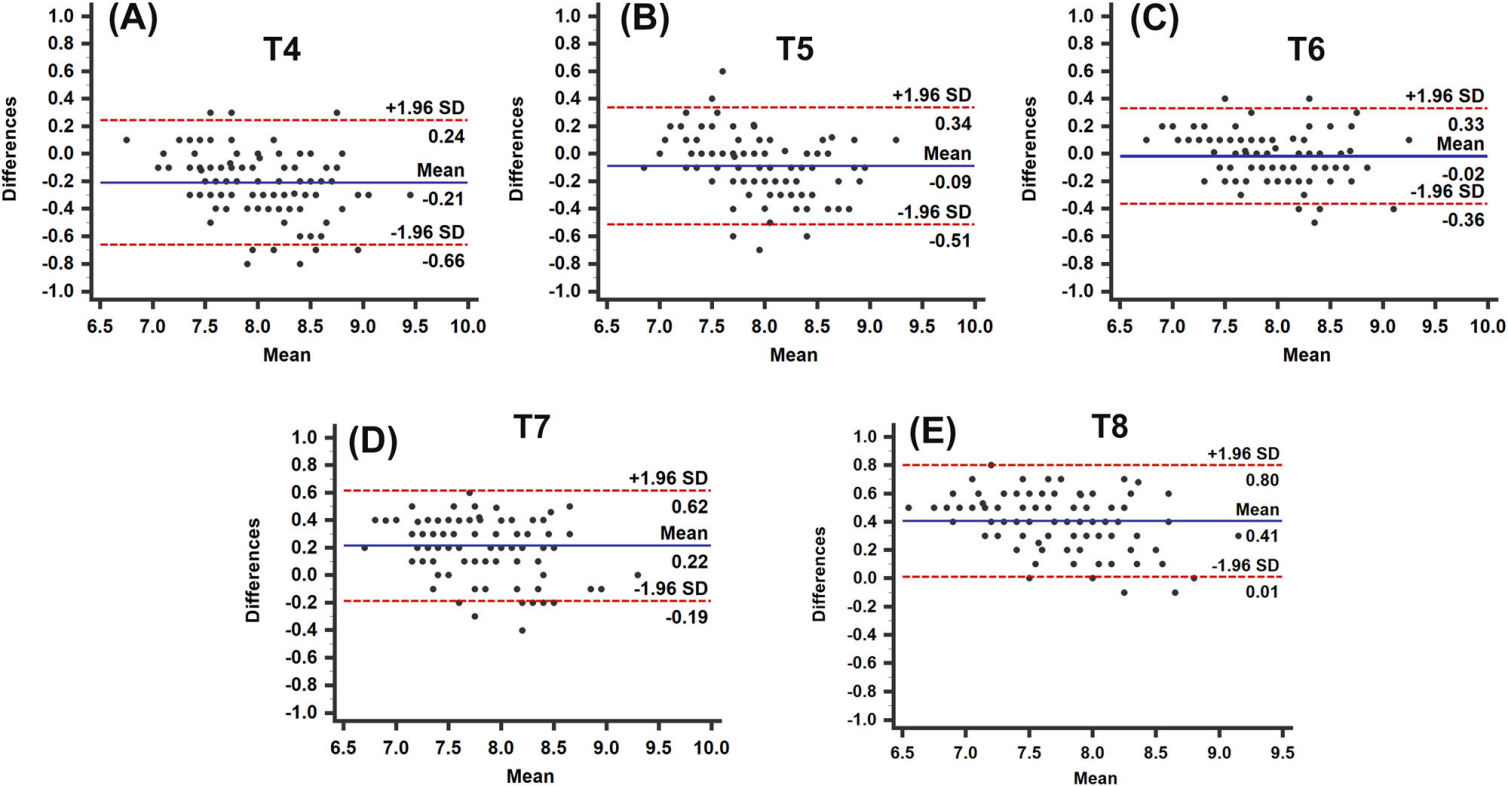
Bland–Altman plots comparing the VHS with the HSVR determined using (A) T4, (B) T5, (C) T6, (D) T7, and (E) T8. The *x*‐axes represent the mean of both measurements, and the *y*‐axes represent the difference between the two measurements. The red dashed lines represent the lower and upper limits of agreement (mean difference ±1.96 SD of the difference). The solid blue lines represent the mean difference. HSVR^T^ *^n^*, heart‐to‐single vertebra ratio determined using the *n*th thoracic vertebra; SD, standard deviation; T*n*, *n*th thoracic vertebra; VHS, vertebral heart size.

The CCC (Figure 2A‒E) showed substantial agreement between the VHS and the HSVR^T6^ (0.95; 95% CI: 0.91–0.97; *p *< .0001; Figure 2C), and moderate agreement between the VHS and the HSVR^T8^ (0.93; 95% CI: 0.90–0.95; *p *< .0001; Figure 2E), HSVR^T5^ (0.92; 95% CI: 0.89–0.95; *p *< .0001; Figure 2B), HSVR^T7^ (0.92; 95% CI: 0.89–0.94; *p *< .0001; Figure 2D), and HSVR^T4^ (0.91; 95% CI: 0.87–0.94; *p *< .0001; Figure 2A). Bland–Altman plots (Figure 3A‒E) for the HSVR versus the VHS showed a bias of −0.023 for the HSVR^T6^ (±0.19; LoA: −0.39 to 0.35; Figure 3C), 0.088 for the HSVR^T5^ (±0.22; LoA: −0.51 to 0.34; Figure 3B), 0.2 for the HSVR^T4^ (±0.23; LoA: −0.66 to 0.24; Figure 3A), 0.22 for the HSVR^T7^ (±0.2; LoA: −0.19 to 0.62; Figure 3D), and 0.41 for the HSVR^T8^ (±0.2; LoA: 0.01‒0.8; Figure 3E). The MAE for the comparison between the HSVR and the VHS was lowest for the HSVR^T6^ (0.14; 95% CI: 0.12–0.17), followed by the HSVR^T5^ (0.18; 95% CI: 0.15–0.21), HSVR^T4^ (0.24; 95% CI: 0.21–0.28), HSVR^T7^ (0.26; 95% CI: 0.23–0.29), and HSVR^T8^ (0.41; 95% CI: 0.37–0.45). The ICC showed excellent interobserver agreement (Table 1; *p *< .001) and good to excellent intraobserver agreement (Table 2; *p *< .001).

**TABLE 1:** Intraclass correlation coefficients for the interobserver agreement among the three observers evaluated on 20 cats.

	ICC (*n* = 20)	95% CI
VHS	0.92	0.48–0.98
HSVR^T4^	0.96	0.85–0.99
HSVR^T5^	0.95	0.79–0.99
HSVR^T6^	0.96	0.79–0.99
HSVR^T7^	0.95	0.78–0.98
HSVR^T8^	0.96	0.73–0.99

Abbreviations: CI, confidence interval; HSVR^T^ *^n^*, heart‐to‐single vertebra ratio determined using the *n*th thoracic vertebra; ICC, intraclass correlation coefficient; VHS, vertebral heart size.

**TABLE 2:** Intraclass correlation coefficients for the intraobserver agreement between the three observers evaluated on 20 cats.

	ICC (95% CI) (*n* = 20)		
	Observer 1	Observer 2	Observer 3
VHS	0.93 (0.81–0.97)	0.87 (0.68–0.95)	0.8 (0.58–0.92)
HSVR^T4^	0.87 (0.7–0.95)	0.88 (0.8–0.94)	0.92 (0.8–0.97)
HSVR^T5^	0.94 (0.63–0.98)	0.88 (0.63–0.95)	0.92 (0.81–0.97)
HSVR^T6^	0.94 (0.83–0.97)	0.89 (0.53–0.96)	0.93 (0.83–0.97)
HSVR^T7^	0.9 (0.7–0.96)	0.9 (0.69–0.96)	0.92 (0.82–0.97)
HSVR^T8^	0.91 (0.78–0.97)	0.75 (0.43–0.9)	0.94 (0.84–0.97)

Abbreviations: CI, confidence interval; HSVR^T^ *^n^*, heart‐to‐single vertebra ratio determined using the *n*th thoracic vertebra; ICC, intraclass correlation coefficient; VHS, vertebral heart size.

## 4 Discussion

This study sought to apply the recently described HSVR method to cats, to assess the level of agreement between this method and the VHS, and to determine the intra‐ and interobserver agreement between the two methods. The results confirmed our hypotheses that the HSVR is a reliable method in cats, demonstrating excellent agreement with the VHS, excellent interobserver agreement, and good to excellent intraobserver agreement.

Objective methods to assess cardiac dimensions, such as the VHS and HSVR, could help clinicians, particularly less experienced ones, detect cardiac disease and determine whether respiratory distress is more likely due to an underlying cardiac disease or a primary respiratory disease [2, 4, 6]. These methods can also contribute to monitoring disease progression and evaluating treatment responses within individual patients [2, 4, 5, 17, 18, 25–28]. The VHS relates the dimensions of the cardiac axes to the thoracic vertebrae, starting from T4, converting and expressing them in tenths of a vertebra [11, 12]. This method assumes that all thoracic vertebral bodies have the same length. Consequently, an alternative approach to calculating the VHS involves dividing the length of the cardiac axes by the length of a single thoracic vertebra, under the assumption of uniform vertebral lengths. However, vertebral body lengths vary not only between subjects but also within the same subject. These variations may arise from true anatomical differences as well as geometric distortions caused by the divergence of the X‐ray beam. In this study, the lengths of vertebral bodies in the T4‐T8 segment were analyzed to identify the vertebra that best represents the average length of all the vertebrae included in this segment. The HSVR offers a value comparable to Litster and Buchanan's VHS [12] but simplifies the process: instead of converting dimensions into vertebral units by counting the number of vertebrae, it relies on a straightforward mathematical division using a single vertebra. This simplification eliminates the need to establish new reference ranges, as existing ranges in the literature remain applicable.

Unlike in dogs, where the HSVR^T7^ showed the best agreement with the VHS, the HSVR^T6^ demonstrated the best agreement with the VHS in cats, showing the lowest mean bias and MAE between the methods. The HSVR^T5^ also showed good agreement, with a low mean bias and MAE. Although the HSVR^T7^ showed good agreement (CCC = 0.92), it had a slightly greater MAE and average bias. Interestingly, the HSVR^T8^ showed good agreement with the VHS but exhibited greater bias. Of the five ratios evaluated, the HSVR^T4^ showed the lowest, although still acceptable, level of agreement with the VHS. These results suggest that the HSVR, particularly HSVR^T6^, can be used as an alternative to the VHS for objective measurement of the cardiac silhouette and should be used in cats with alterations of the thoracic vertebral bodies or cats with excessive lordosis of the thoracic spine. Modifications of the thoracic vertebral column (e.g., spondylosis deformans), reduced intervertebral disc space, or shortening of the thoracic vertebral column length due to the lordosis that cats tend to assume during the acquisition of thoracic radiographs, particularly in unsedated patients, can cause a real or apparent shortening of the thoracic vertebral column that hinders correct measurement and may lead to overestimation of the VHS. In contrast, the HSVR considers the length of a single vertebral body between T4 and T8, and it is less affected by these factors.

The HSVRs showed excellent interobserver agreement because all values were at least 0.95. Of note, for the interobserver agreement, all the HSVRs showed a higher ICC than the VHS (Table 1). This result is remarkable because the VHS has been routinely used for several years in our clinic, and Observers 1 and 2 work in close contact daily and perform the measurements similarly, whereas the HSVR is a newer method that has been utilized for a shorter period of time. Furthermore, all the observers followed the same written and illustrated instructions, thus reducing the possible sources of variability when measuring the parameters. This result is likely to be related to the easier method of calculating the cardiac silhouette dimensions using the HSVR method without the need to transpose the cardiac axes over the thoracic spine or span the conversion of the cardiac axis in vertebral units, reducing the method's susceptibility to interobserver variability [6, 19, 29]. The good to excellent intraobserver agreement with similar ICC values among observers and between the VHS and HSVRs is ascribed to the same reasons.

In the current study, the presence or absence of an underlying cardiac disease was not considered to increase the sample's randomness, making it more representative of the cat population in clinical settings. Furthermore, we decided to exclude cats aged <12 months to avoid erroneous results due to the disproportionate ratio between the cardiac axes and the vertebral length. Indeed, in previous studies, the VHS was significantly greater in healthy, skeletally immature cats than in adult cats, likely due to the larger heart size relative to other immature skeletal structures, such as the thoracic vertebrae, in younger cats [28, 30]. We also excluded cats with sternal alterations, particularly pectus excavatum, because these alterations deform the cardiac silhouette, hindering correct evaluation of the cardiac silhouette axes. We did not consider the body condition score because a previous study suggested that the VHS is minimally affected by intrathoracic fat deposits [13].

A limitation of this study is the relatively high experience of the three observers, and two of them (Observers 1 and 2) work in daily close contact. Although the measurements used to determine the inter‐ and intraobserver agreement were performed independently in this study, this approach could have increased the agreement between their measurements. A previous study investigating the VHS interoperator agreement demonstrated that while the method is independent of observer experience, it is mainly influenced by individual observers' selection of reference points and the transformation of long and short‐axis dimensions into VHS units [6]. Since the HSVR method eliminates the need for one of these steps (i.e., the transformation of long and short‐axis dimensions into VHS units), it is deemed to be an easier and more reliable method to apply. Further multicentric studies, including groups of observers with varying degrees of expertise, should investigate this aspect. Another limitation is the high proportion of domestic short‐hair cats and the small proportion of purebred cats included in the final sample. However, this likely represents the clinical setting in our geographical area. Furthermore, the thoracic and cardiac morphology of cats is less affected by interbreed variability than is the case for dogs [7, 14, 15, 31].

In conclusion, HSVR is a simple, reliable modality for assessing the cardiac silhouette size in cats, and the best agreement was found between the VHS and the HSVR^T6^. The HSVR also showed excellent interobserver agreement and good to excellent intraobserver agreement.

## List of Author Contributions

### Category 1

(a) Conception and Design: Costanza, Meomartino.
(b) Acquisition of Data: Meomartino, Castiello, Coluccia, Sangiuliano, Greco, Piantedosi, Costanza.
(c) Analysis and Interpretation of Data: Costanza, Meomartino, Navas.

### Category 2

(a) Drafting the Article: Costanza, Meomartino.
(b) Reviewing Article for Intellectual Content: Greco, Piantedosi, Coluccia, Castiello, Sangiuliano, Navas.

### Category 3

(a) Final Approval of the Completed Article: Costanza, Greco, Piantedosi, Castiello, Coluccia, Sangiuliano, Navas, Meomartino.

### Category 4

(a) Agreement to be accountable for all aspects of the work in ensuring that questions related to the accuracy or integrity of any part of the work are appropriately investigated and resolved: Costanza, Greco, Piantedosi, Castiello, Coluccia, Sangiuliano, Navas, Meomartino.

No third‐party support was received in connection with this study or in the writing or publication of the manuscript.

## Declaration

No third‐party support was received in connection with this study or the writing or publication of the manuscript.

## Conflicts of Interest

The authors declare no conflicts of interest.

## Previous Presentation or Publication Disclosure

Preliminary results of this study were presented as oral communication at the EVDI Annual Congress, 18‒21 September 2024, Athens, Greece.

## Reporting Checklist Disclosure

The authors followed the GRRAS checklist for reporting reliability and agreement studies.

## Data Availability

Data are available from the corresponding author upon reasonable request.
